# A quantitative metabolomics profiling approach for the noninvasive assessment of liver histology in patients with chronic hepatitis C

**DOI:** 10.1186/s40169-016-0109-2

**Published:** 2016-08-18

**Authors:** M. Omair Sarfaraz, Robert P. Myers, Carla S. Coffin, Zu-Hua Gao, Abdel Aziz M. Shaheen, Pam M. Crotty, Ping Zhang, Hans J. Vogel, Aalim M. Weljie

**Affiliations:** 1 Department of Biological Sciences, University of Calgary, 2500 University Drive, North West, Calgary, AB T2N 1N4 Canada; 2 Department of Medicine-Pathology and Molecular Medicine, McMaster University, Hamilton, ON L8N 3Z5 Canada; 3 Liver Unit, Division of Gastroenterology and Hepatology, Cumming School of Medicine, University of Calgary, Hospital Drive North West, Calgary, AB T2N 4Z6 Canada; 4 Department of Pathology, McGill University Health Centre, 1001 Decarie Boulevard, Montreal, QC H4A 3J1 Canada; 5 Liver Unit, Teaching and Wellness Building, University of Calgary, Hospital Drive, North West, Calgary, AB T2N 4Z6 Canada; 6 Department of Chemistry, University of Calgary, 2500 University Dr. NW Calgary, Calgary, AB T2N 1N4 Canada; 7 Metabolomics Research Center, Department of Biological Sciences, University of Calgary, Calgary, AB T2N 1N4 Canada; 8 Department of Pharmacology, University of Pennsylvania, Philadelphia, PA 19104 USA; 9 Department of Systems Pharmacology and Translational Therapeutics, Perelman School of Medicine, University of Pennsylvania, Philadelphia, PA 19081 USA; 10 Dept. of Medicine/Dept. of Pathology and Molecular Medicine, McMaster University, 1280 Main Street West, Health Sciences Centre, Hamilton, ON L8S4K1 Canada

**Keywords:** Hepatitis C, Metabolomics, ^1^H-NMR spectroscopy

## Abstract

**Background:**

High-throughput technologies have the potential to identify non-invasive biomarkers of liver pathology and improve our understanding of basic mechanisms of liver injury and repair. A metabolite profiling approach was employed to determine associations between alterations in serum metabolites and liver histology in patients with chronic hepatitis C virus (HCV) infection.

**Methods:**

Sera from 45 non-diabetic patients with chronic HCV were quantitatively analyzed using ^1^H-NMR spectroscopy. A metabolite profile of advanced fibrosis (METAVIR F3-4) was established using orthogonal partial least squares discriminant analysis modeling and validated using seven-fold cross-validation and permutation testing. Bioprofiles of moderate to severe steatosis (≥33 %) and necroinflammation (METAVIR A2-3) were also derived. The classification accuracy of these profiles was determined using areas under the receiver operator curves (AUROCSs) measuring against liver biopsy as the gold standard.

**Results:**

In total 63 spectral features were profiled, of which a highly significant subset of 21 metabolites were associated with advanced fibrosis (variable importance score >1 in multivariate modeling; R^2^ = 0.673 and Q^2^ = 0.285). For the identification of F3–4 fibrosis, the metabolite bioprofile had an AUROC of 0.86 (95 % CI 0.74–0.97). The AUROCs for the bioprofiles for moderate to severe steatosis were 0.87 (95 % CI 0.76–0.97) and for grade A2–3 inflammation were 0.73 (0.57–0.89).

**Conclusion:**

This proof-of-principle study demonstrates the utility of a metabolomics profiling approach to non-invasively identify biomarkers of liver fibrosis, steatosis and inflammation in patients with chronic HCV. Future cohorts are necessary to validate these findings.

**Electronic supplementary material:**

The online version of this article (doi:10.1186/s40169-016-0109-2) contains supplementary material, which is available to authorized users.

## Background

An estimated 185 million people worldwide are chronically infected with the hepatitis C virus (HCV) [1]. Chronic HCV carriers are at risk of progressive liver fibrosis, and up to 30 % can develop cirrhosis after decades of infection with significant risk of morbidity and mortality. Hepatic fibrosis occurs in the setting of liver injury from immune mediated chronic inflammation and hepatocyte cell turnover. This can ultimately lead to cirrhosis, manifested by architectural distortion of the liver and nodule formation [2]. Accumulation of the extracellular matrix occurs following metabolic and synthetic impairment of hepatocytes.

Recently the HCV infection treatment landscape has changed with the advent of new oral directly acting antiviral agents which offer >90 % successful treatment response rates in patients with HCV genotype 1 infection. Successful treatment is defined as a sustained virological response, SVR or undetectable HCV RNA by sensitive PCR assay 3 months after the end of anti-HCV treatment [3, 4]. The achievement of an SVR has been shown to correlate with reduced risk of liver disease progression and even reversal of liver fibrosis development. Thus, the currently approved antiviral therapy is recommended in all patients with advanced fibrosis or cirrhosis who have the highest risk of HCV-related complications such as liver failure and hepatocellular carcinoma. However, treatment is costly and not widely available, especially in resource-limited countries. Thus accurate fibrosis staging, especially the diagnosis of cirrhosis, is needed in clinical practice to determine the urgency and need for anti-HCV antiviral therapy [5, 6].

The current gold standard for the diagnosis HCV-related fibrosis and cirrhosis is liver biopsy. However biopsy is limited due to risk, invasive nature, technical issues (i.e., sample size, sampling error) and patient willingness. A number of complementary non-invasive tools have been developed and validated to diagnose and monitor liver fibrosis progression. The “physical approach” is characterized by the measurement of liver stiffness by diagnostic or ultrasound imaging techniques (i.e., Acoustic Radiation Force Impulse ultrasound imaging, and transient elastography; by FibroScan [7–11]. The “biological approach” focuses on serum biomarkers as well as developing profiles using high-throughput technologies such as genomics, transcriptomics, glycomics and metabolomics. Serum biomarkers include inexpensive tests such as the AST-to-Platelet Index (APRI), as well as the commercially developed FibroTest). Shaheen et al. reported that APRI under 0.5 could exclude significant fibrosis with 80 % accuracy [12]. It was however limited in accurately staging fibrosis in about 65 % of the patients in the study. Poynard et al. assessed the performance of Fibrotest and reported and area under the receiver operating characteristic curve of 0.80 (95 % confidence interval 0.78–0.82) for the identification of significant fibrosis (Stage 2–4) [13]. These various tests may also be limited by sensitivity, specificity, as well as cost and availability. Consequently, there remains a need for complementary non-invasive biomarkers to assist in treatment decisions and to monitor the progression or resolution of liver fibrosis.

Metabolomics as a high-throughput platform offers to capture the metabolic alterations that are reflective of the disease process. For example, we have successfully characterized gastrointestinal tumor characteristics using serum metabolic profiles [14, 15]. Since the liver is involved in important metabolic processes, pathological process such as hepatic fibrosis manifests as metabolic alterations that can assist in understanding the disease processes. For example, in a recent study, Jimenez et al. were able to characterize serum metabolites using ^1^H-NMR metabolomics in cirrhotic patients at risk of developing hepatic encephalopathy [16]. Recently, Baniasadi et al. were able to discriminate between cirrhotic HCV patients who were at high risk for developing hepatocellular carcinoma (HCC), from those who had already progressed to HCC using blood metabolite measurements [17]. Gao et al. were able to distinguish healthy controls when compared with serum samples from patients with liver cirrhosis and HCC [18], while HCV patients pre-therapy have been shown to have elevated levels of tryptophan in patients with SVR compared to those who were non-responders to antiviral therapy [19].

Despite this work in clinical populations, there is limited information specifically regarding the association of liver histology with clinically accessible metabolic profiles from blood or urine in HCV populations, the exception being a study examining HIV/HCV co-infection [20]. In the current study, patients with chronic HCV infection (>80 % genotype 1 infection) with varying degrees of fibrosis (F0–4), necroinflammation (A1–3) and steatosis were evaluated. We hypothesized that a metabolomics approach would be able to stratify HCV related liver histological disease progression by analysis of patient sera samples using a quantitative NMR metabolomics profiling approach. Using multivariate statistical analysis tools, we could identify non-invasive metabolite biomarkers that are capable to discriminate between various stages of fibrosis, necroinflammation and steatosis.

## Methods

### Study design

The study included patients chronically infected with the HCV seen at the Calgary Liver Unit, Viral Hepatitis Clinic, Department of Gastroenterology and Hepatology, University of Calgary. Study inclusion criteria included (1) adult patients (≥18 years of age,) (2) presence of chronic HCV infection (positive anti-HCV antibodies and HCV RNA in serum), (3) biopsy diagnosis of fibrosis and necroinflammation, according to METAVIR classification for HCV [21], (4) macrovesicular steatosis assessment by histopathology and graded as none (0–5 %), mild (5–32 %), moderate (33–65 %) and severe (≥66 %) steatosis. The exclusion criteria included: (1) obvious cirrhosis based on radiological investigations (e.g. nodular liver, splenomegaly), or clinical features of decompensated liver disease (e.g. jaundice, encephalopathy, ascites), (2) conditions that may alter the accuracy of biomarkers of fibrosis including extrahepatic obstruction, immunosuppression (e.g. due to concomitant HIV infection, medication), pregnancy, and systemic inflammatory conditions (e.g. sepsis, inflammatory bowel disease) [22], (3) excessive alcohol consumption defined as ≥40 g/d for men and ≥20 g/d for women in patients with HCV, (4) antiviral therapy for HCV within the previous 6 months. The study approval was obtained from the University of Calgary Conjoint Health Research Ethics Board (CHREB) and all study participants provided signed informed consent for study enrolment according to the Declaration of Helsinki. Relevant C = clinical and demographic information collected included, gender, age, HCV genotype, serum alanine-aminotransferase (ALT) levels, and body mass index (BMI).

Peripheral blood samples were collected approximately 24 h after liver biopsy was performed, after a 12-h fast. The isolated serum were immediately transferred to cryovials and stored at −80 °C for subsequent metabolomics analysis. In total 45 study participants with chronic HCV infection were enrolled, for sera spectra analysis. Participants were divided into three groups based on liver histopathology characteristics of fibrosis, necroinflammation and steatosis (Table 1).

**Table 1:** Patient demographics and biopsy characteristics of HCV patients with fibrosis (F0–4) and necroinflammation (A1–3) based on METAVIR scoring system

Patient characteristics	
Age, years	46 (18–60)
Male gender	67 %
HCV genotype 1	80 %
ALT, U/L	147 (25–478)
BMI, kg/m^2^	26.7 (17.6–53.7)
Fibrosis (F0–4), %	
F0	9
F1	20
F2	38
F3	13
F4	20
Necroinflammation (A1–3), %	
A1	20
A2	38
A3	4
Biopsy quality	
Length, cm	2.0 (0.9–3.9)
Number of portal triads	14 (3–29)

### Liver tissue sampling and histopathology evaluation

Percutaneous liver biopsy was performed under local anesthesia with an ultrasound guidance via the right coastal approach [23]. An 18 gauge (width 1.2 mm, cutting depth 2.2 cm) spring loaded, cutting needle (Bard^®^, Monopty^®^ Percutaneous biopsy instrument; C.R. Bard-Inc., Billerica, MA) was used. If a sample less than 2.0 cm long was obtained after the first pass of the needle, an additional pass was performed. Following biopsy, patients were observed according to a standardized, 4 h protocol to observe for immediate complications [23]. The liver biopsy tissue was fixed, paraffin-embedded and stained with hematoxylin and eosin and Massaon’s trichome. All biopsies were examined by two hepatopathologists blinded to the clinical data. The size of biopsy, number of portal triads and fragmentation were recorded to account for the effect of these factors on the accuracy of the biomarkers under study [24, 25]. Liver fibrosis was staged from 0 to 4 according to the METAVIR classification [21] and NALFD score [22]. Histopathological assessment of fibrosis and necroinflammation were done using the METAVIR grading system. These classifications have excellent intra- and inter-observer agreement for liver fibrosis (kappa values >0.80) [21, 22].

The METAVIR scoring system assesses fibrosis in chronic HCV patients in accordance to a 5-stage classification, as previously published. Based on the percentage of involved hepatocytes, steatosis were divided into 4 groups as none (0–5 %), mild (5–32 %), moderate (33–65 %) and severe (≥66 %) steatosis.

## ^1^H NMR spectroscopy

^1^H NMR spectroscopy was performed using a protocol previously described [26]. For NMR analysis, serum samples were thawed on ice. 250 μl of serum sample were filtered through a prewashed Nanasep 3 K Omega Filter Eppendorf to remove high molecular weight (>3 kDa) compounds (e.g. large proteins, lipid complexes etc.). The filtrate was then centrifuged and buffered to a pH of 7.0 for analysis. Regular one-dimensional proton NMR spectra were obtained using a 600-MHz Bruker Ultrashield NMR spectrometer (Bruker Biospin, Milton, Canada). The spectra were acquired using a standard NOESY 1D pulse sequence that had good water suppression characteristics and is commonly used for metabolite profiling of serum samples [27]. Relaxation delay of 1 s was used; t1 was set to 4 μs and a 100 ms mixing time was employed. Initial samples for each batch were shimmed to ensure half-height line width of <1.1 Hz for the dimethyly-silapentane-sulphonate peak, calibrated to 0.0 ppm. Spectra were acquired with 1024 scans, then zero filled and Fourier transformed to 128 k data points using the Chenomx NMRSuite processor. Additional 2-dimensional NMR experiments were performed for the purpose of confirming chemical shift assignments, including homonuclear total correlation spectroscopy (2D ^1^H-^1^H TOCSY) and heteronuclear single quantum coherence spectroscopy (2D ^1^H-^13^C HSQC), using standard Bruker pulse programs.

### Data processing for statistical analysis

Raw data from ^1^H NMR was processed and profiled using ChenomX NMR Suite software 4.6 (Cheonomx Inc., Edmonton, Canada) to a library of 63 compounds. ^1^H-NMR spectral data was evaluated using the strategy of “targeted profiling” [28]. This allowed quantification of metabolite concentrations in the serum samples. The data was subsequently log transformed, centered and unit variance scaled. Data analysis was done using multi-variate statistical analytical software SIMCA- P + (V12, Umetrics AB, Umea, Sweden). Firstly, a principal component analysis (PCA) was performed to detect any group separation based on variation in NMR signals. PCA was also performed to check the unsupervised segregation of the metabolome.

Orthogonal projection least squares-discriminant analysis (OPLS-DA) was done which allowed us to differentiate between the variables concerning the different stages of fibrosis. Model variance and predictive ability was assessed using R^2^ and Q^2^ values. The R^2^X and R^2^Y values were representative of the explained variation in the X and Y matrices respectively. The Q^2^Y value was indicative of the predictability of the model generated. Model significance was assessed using a cross-validated ANOVA based on seven-fold cross validation. Variables were selected according to the VIP (variables importance in projection) values, which were reflective of the correlation of the metabolites towards different response. Model performance was assessed using AUROC (area under the receiver operator curve).

### Pathway analysis

Preliminary metabolite function was assigned using the Human Metabolome Database [29]. Dataset of metabolites that were differentially abundant in the NMR analysis were uploaded into the program. This was done independently for each comparison. Metabolite Set Enrichment Analysis (MSEA) [30] was used to elucidate the various biochemical pathways involved in HCV fibrosis, necroinflammatory disease and steatosis.

## Results

### Patients

In total 45 study participants with chronic HCV infection were enrolled, for sera spectra analysis. Participants were divided into three groups based on liver histopathology characteristics of fibrosis, necroinflammation and steatosis (Table 1). These included participants who had fibrosis and necroinflammation (classified according to the METAVIR score [21] and patients with moderate to severe steatosis. One-third of the patients were scored with F3–4 fibrosis; 13 and 42 % had moderate to severe steatosis and inflammation, respectively.

### Metabolomic analysis

#### Metabolite bioprofile of advanced HCV fibrosis (F3–4)

**Fig. 1:**
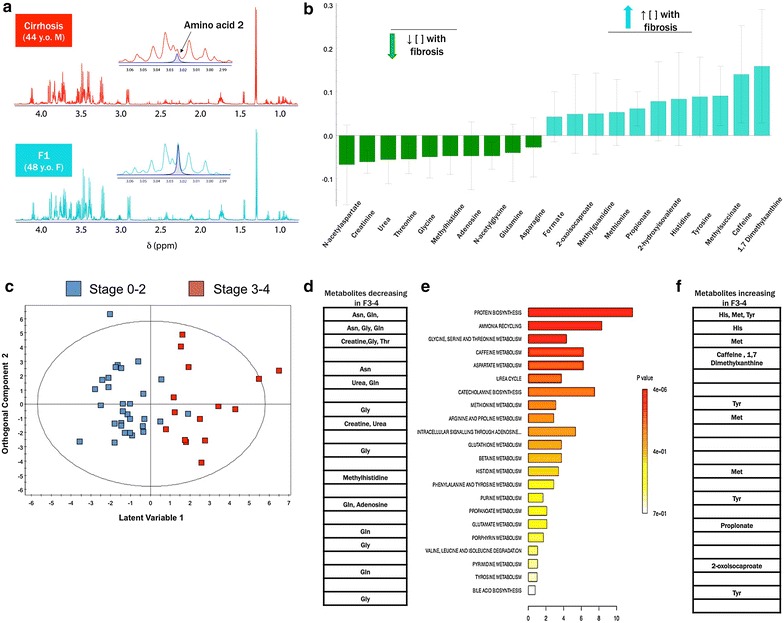
Metabolite bioprofiling facilitates discrimination of advanced HCV fibrosis (F3–4). **a** Illustrates the relative decrease in concentration of creatine in HCV carrier with F4 fibrosis (cirrhosis) in comparison with a HCV carrier with Stage 1 (F1) liver fibrosis in the NMR spectra. **b** Represents the coefficient plot from the OPLS-DA analysis showing differences in serum metabolite concentration in the patients with HCV fibrosis (F3–4). **C** Shows OPLS-DA score plots of serum samples from HCV patients with fibrosis. Each data point is representative of the complete metabolite measurement from one HCV patient: *blue square* Stage 0–2, *red square* Stage 3–4. The *x* and *y-axis* represent the latent variable 1 and orthogonal component 2 respectively. R^2^ is the explained variance; Q^2^ is the predictive ability of the model; Model significance was assessed using a cross-validated ANOVA based on seven-fold cross validation (R^2^ = 0.673, Q^2^ = 0.285, p = 0.008). Biochemical pathways involved in HCV advanced fibrosis (F3–4). **d** Illustrates the metabolites that have been relatively decreased in the fibrosis group. **e** Indicates the biochemical pathways involved in fibrosis model; higher intensity with a higher association with the fibrotic group. **f** Represents the metabolites which have been relatively increased in the fibrotic group

For ^1^H NMR spectral profiling, a total of 63 features were profiled. The results indicated that 21 metabolites were differentially abundant associated with advanced fibrosis (variable importance score >1 in multivariate modeling) (Table 2). The ^1^H NMR spectra of two patient’s sera, with F4 (cirrhosis) and F1 stage of fibrosis are represented in Fig. 1a. It illustrates the relative decrease in the concentration of creatine in patients with cirrhosis (F4) in comparison with patients with F1 fibrosis, supporting the results indicated in the coefficient plot (Fig. 1b). The sera spectra of patients with F0-2 and F3-4 were well discriminated with the OPLS-DA model (R^2^ = 0.673) (Fig. 1c). The predictive ability of the model was measured by sevenfold cross validation (Q^2^ = 0.285), and cross-validated ANOVAl indicates a significant model (p = 0.008). Figure 1e is reflective of biochemical pathways over-represented in advanced fibrosis, with metabolites shown in Fig. 1d being relatively decreased as a function of increasing fibrosis, while those listed in Fig. 1f have been relatively increased.

**Table 2:** Chemical classes and the associated metabolites identified with respect to the HCV patients with fibrosis (F3–4), necroinflammation (A2–3) and steatosis (≥33 %)

Outcome [#spectral features]	Chemical classes (n)	Metabolites
Fibrosis (F3–4) [21]	Acyl Glycine (1)	*N*-acetylglycine
	Amino acids (10)	Asparagine, creatine, glutamine, glycine, histidine, methionine, methylhistidine, *N*-acetylaspartate, threonine, tyrosine
	Amino ketones (1)	Urea
	Dicarboxylic acid (1)	Methylsuccinate
	Fatty acids (2)	Formate, propionate
	Hydroxy acids (1)	2-Hydroxyisovalerate
	Keto-acids (1)	2-Oxoisocaproate
	Nucleoside Analogue (1)	Adenosine
	Polyamines (1)	Methylguanidine
	Purine/purine deivatives (2)	1,7-Dimethylxanthine, Caffeine
Necroinflammation (A2–3) [17]	Acyl glycine (1)	*N*-Acetylglycine
	Alcohols (1)	Ethanol
	Aliphatic and aryl amines (1)	Dimethylamine
	Amino acids (6)	Creatine, histidine, glutamate, phenylalanine, tryptophan, tyrosine
	Dicarboxylic acid (3)	Methylsuccinate, suberate, succinate
	Quaternary amines (1)	*O*-acetylcarnitine
	Nucleoside analogue (1)	Adenosine
	Purine/purine derivatives (2)	1,7-Dimethylxanthine, caffeine
	Tricarboxylic acid (1)	Citrate
Steatosis (≥33 %) [16]	Amino acids (6)	Asparagine, creatine, creatinine, L-glutamate, SERINE, tryptophan
	Carbohydrates (1)	d-Mannose
	Dicarboxylic acid (4)	2-Oxoglutarate, methylsuccinate, suberate, succinate
	Hydroxy acids (2)	3-Hydroxybutyrate, lactate
	Keto-acids (2)	Pyruvate
	Ketones (1)	Acetone
	Nucleoside analogues (1)	Adenosine

#### Metabolite bioprofiles of necroinflammation (A2–3) and steatosis (>33 %)

**Fig. 2:**
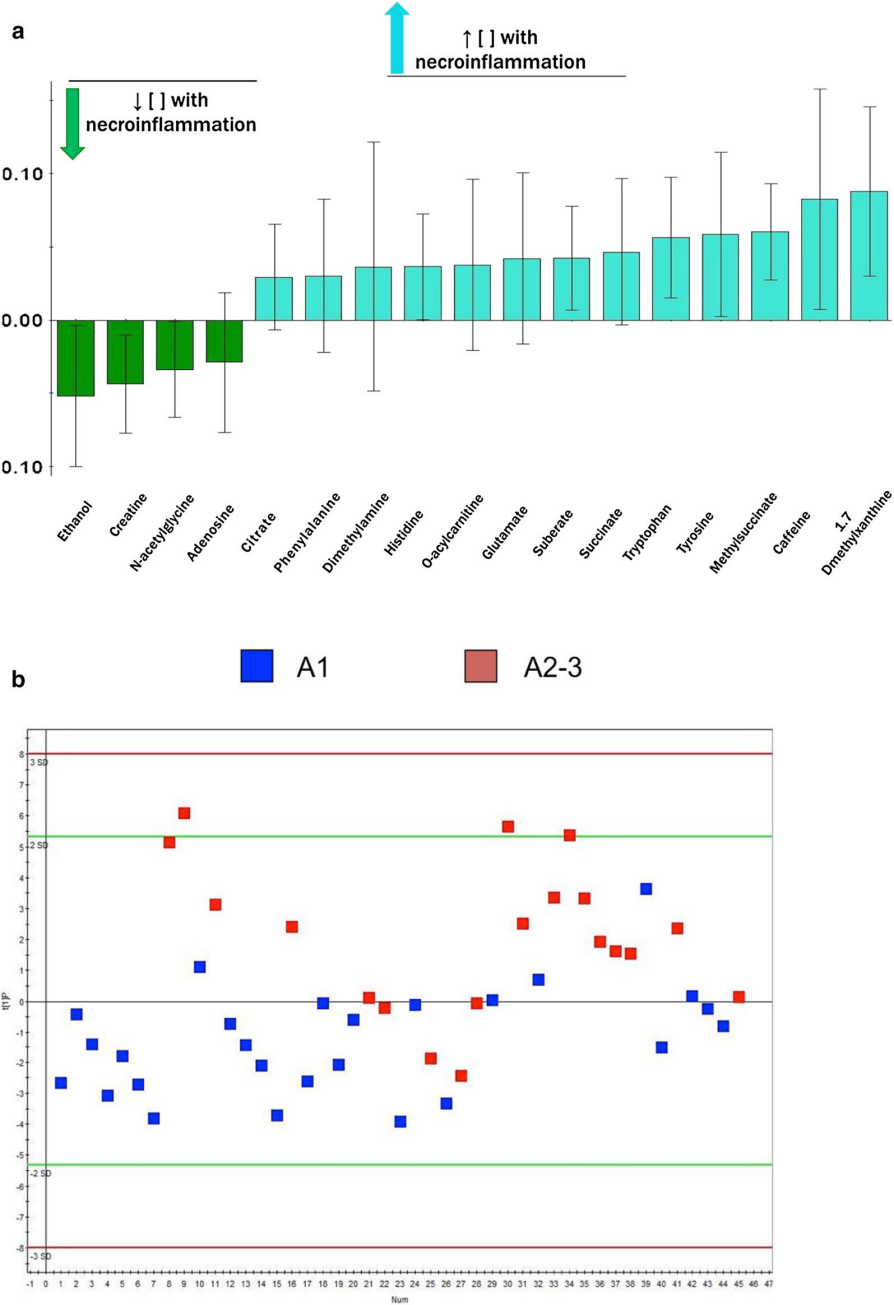
Metabolite bioprofiling facilitates discrimination of HCV necroinflammation (A2–3). **a** Represents the coefficient plot from the OPLS-DA analysis showing differences in serum metabolite concentration in the patients with necroinflammatory disease (A2–3). **b** Shows OPLS-DA score plots of serum samples from HCV patients with necroinflammation. Each data point is representative of the complete metabolite measurement from one HCV patient: *blue square* A1, *red square* A2–3. The t[1] value represent the score of each sample in principal component 1. R^2^ is the explained variance; Q^2^ is the predictive ability of the model; Model significance was assessed using a cross-validated ANOVA based on seven-fold cross validation (R^2^ = 0.405, Q^2^ = 0.102, p = 0.10)

Based on ^1^H-NMR profiling, patients with necroinflammatory activity classified as grade A2 and A3 (moderate to severe activity) could be partially distinguished from patients graded as A1 (minimal activity). The sera of patients with necroinflammation (A2–3) were distinguished from the rest of the necroinflammatory samples, based on differences in 17 spectral features (Table 2) in an OPLS-DA model with a variation of R^2^ = 0.405, Q2 = 0.102 and was weakly significant CV-ANOVA p = 0.10 (Fig. 2b). Of these metabolites that were identified, eight were shared with the fibrosis model (Table 3). The coefficients plot representing the differential abundance for each of the metabolites is illustrated in Fig. 2a.

**Table 3:** The commonalities in metabolite bioprofiles of fibrosis (F3–4), steatosis (≥33 %) and necroinflammation (A2–3)

Steatosis (≥33 %) and fibrosis (F3–4) N = 4	Fibrosis (F3–4) and necroinflammation (A2–3) N = 8	Necroinflammation (A2–3) and steatosis (≥33 %) N = 7
Asparagine Creatine Methylsuccinate Adenosine	N-acetylglycine Creatine Histidine Tyrosine Methylsuccinate Adenosine 1,7-Dimethylxanthine Caffeine	Creatine Glutamate Tryptophan Methylsuccinate Suberate Succinate Adenosine

**Fig. 3:**
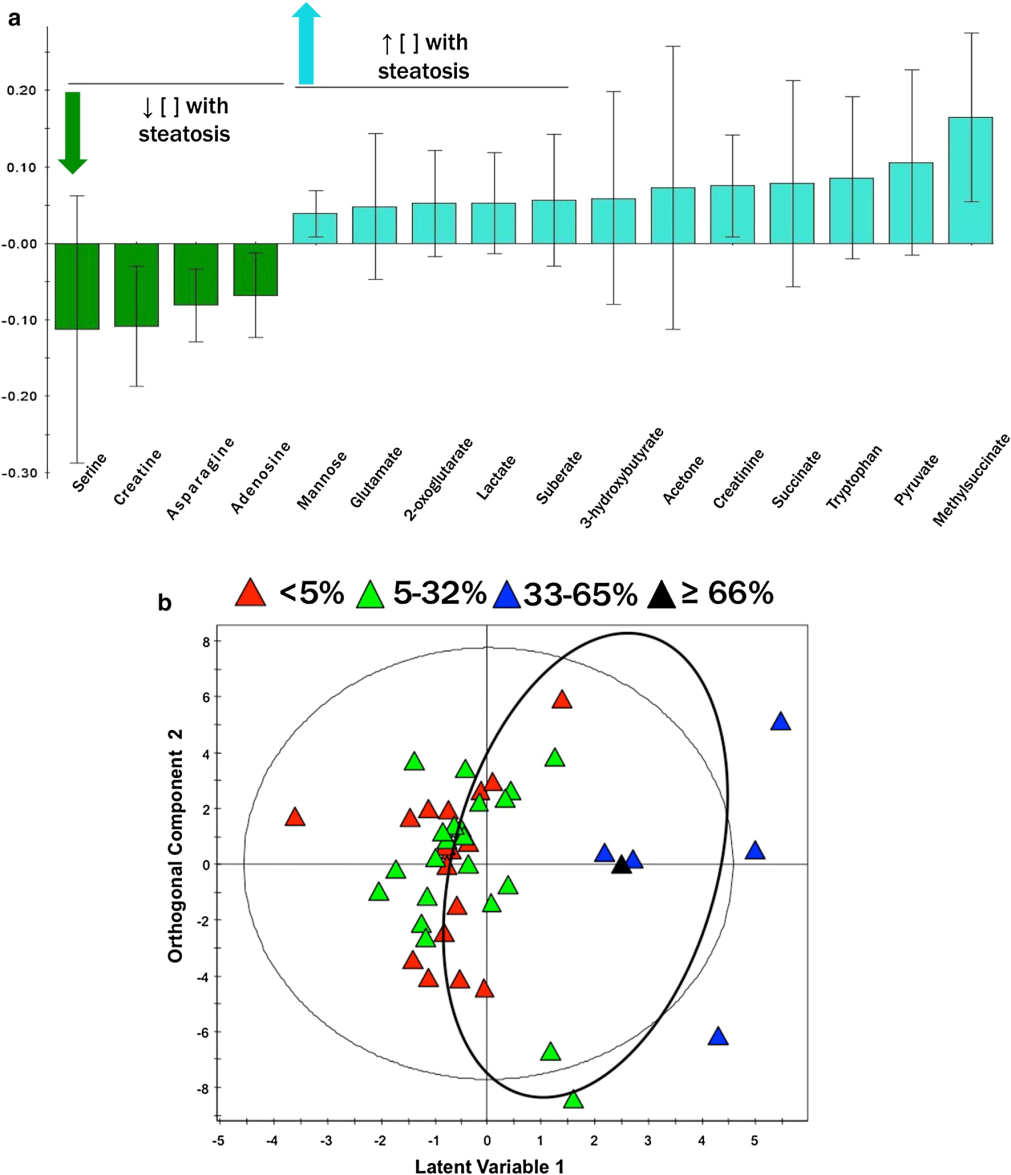
Metabolite bioprofiling facilitates discrimination of HCV patients with steatosis (≥33 %). **a** Represents the coefficient plot from the OPLS-DA analysis showing differences in serum metabolite concentration in the patients with steatosis. **b** Shows OPLS-DA score plots of serum samples from HCV patients with steatosis. Each data point is representative of the complete metabolite measurement from one HCV patient: *red triangle* <5 %, *green triangle* 5–32 %, *blue triangle* 33–65 %, and *black triangle* ≥66 %. The *x* and *y-axis* represent the latent variable 1 and orthogonal component 2 respectively. R^2^ is the explained variance; Q^2^ is the predictive ability of the model; Model significance was assessed using a cross-validated ANOVA based on sevenfold cross validation (R^2^ = 0.67, Q^2^ = 0.16, p = 0.11)

Study participants were divided into 4 categories based on degree of liver steatosis (<5, 5–32, 33–65 and ≥66 %). A distinction in-group with ≥33 % was observed in OPLS-DA with a variation of R^2^ Y = 0.67, Q2Y = 0.166 and weakly significant with a CV-ANOVA = 0.11 (Fig. 3b). Among the sixteen metabolites that were found to correspond to the patient sera samples with a steatosis of ≥33 %, four of those metabolites were common with the fibrosis model whereas seven were shared with the necroinflammation model (Table 3). Metabolites derived from the model and their respective differential abundance are illustrated in Fig. 3a.

### Clinical applicability of the fibrosis, necroinflammation and steatosis model

**Fig. 4:**
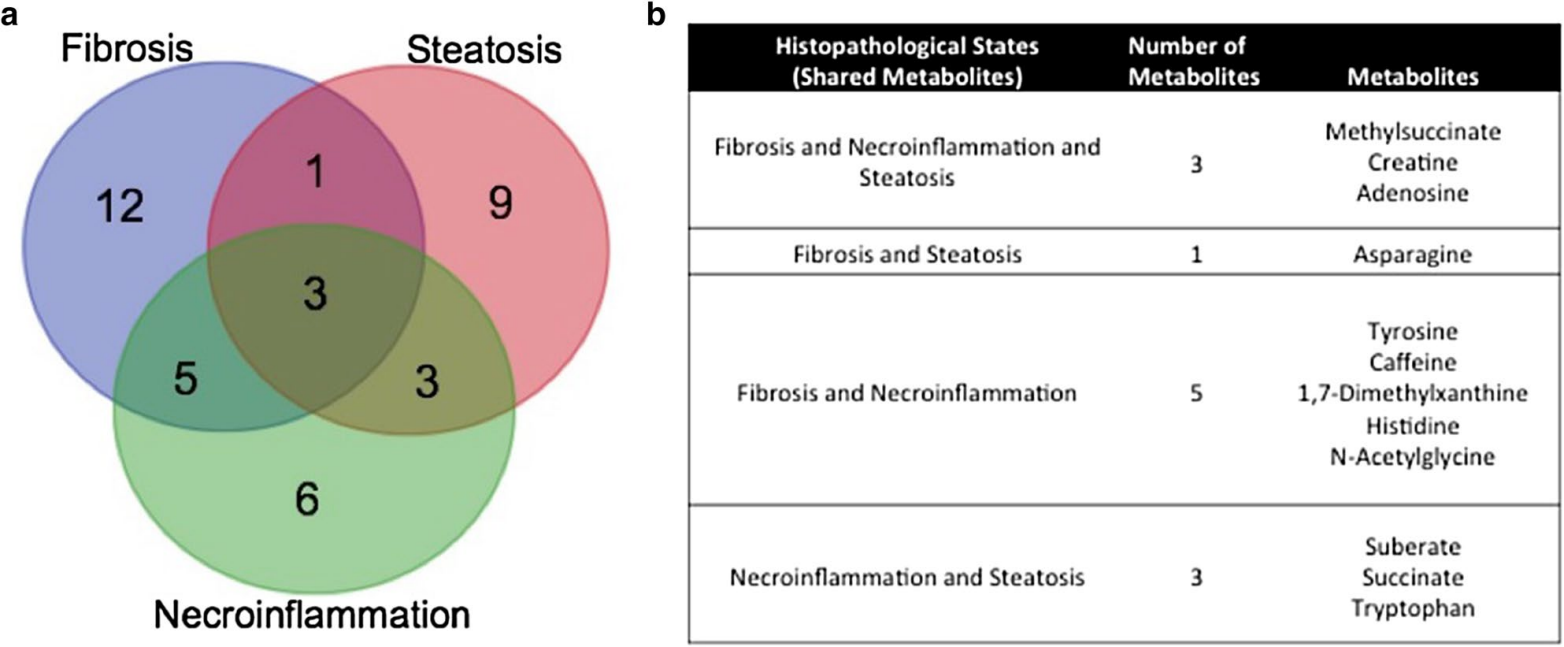
Overall significant metabolites from models of fibrosis, necroinflammation and steatosis. **a** *Venn diagram* illustrating degree of overlap between metabolites changed with highest significance (VIP > 1) in each model. **b** Summary of metabolites by overlapping group

As an assessment of the possible applicability of the metabolites from each pathophysiological model, the relative overlap between metabolites from each model was assessed (Fig. 4). Of the total set of metabolites deemed to be discriminating in each case, three metabolites were shared (methylsuccinate, creatine, and adenosine). In addition, asparagine was shared between the fibrosis and steatosis models. A listing of the overlapping metabolites is provided in Fig. 4b. Notably, a large subset of metabolites were unique to each model; twelve metabolites were discriminatory only in the fibrosis testing. This result is encouraging as it suggests that clinical applicability is truly based on metabolic differences.

We further tested the predictive performance of discriminant model between sera of patients with stages F0–2 and F3–4, A0–1 and A2–3 and steatosis (≥33 %) respectively. This was done by constructing seven models with one-seventh of the data excluded from each of the seven models, with each sample excluded once. This method provided the predictive ability of the model.

**Fig. 5:**
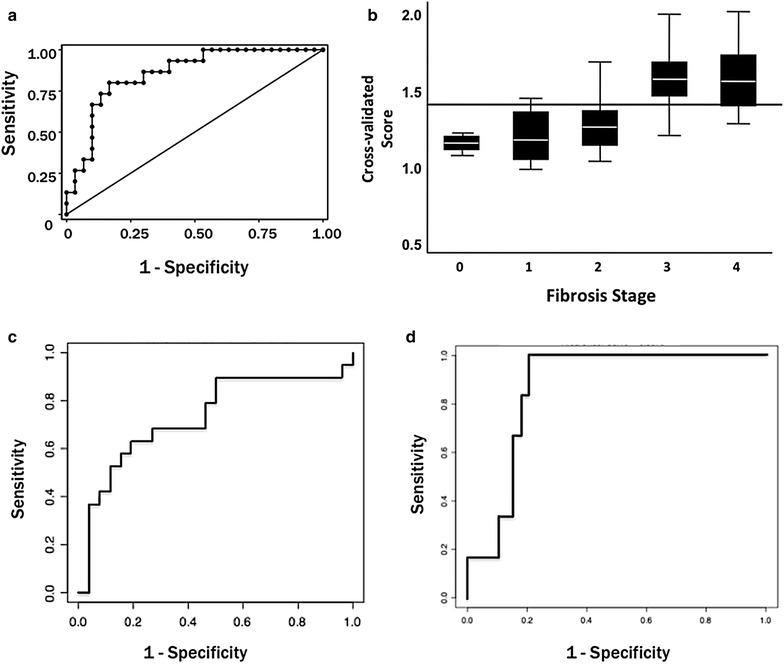
Clinical applicability of the advanced fibrosis (F3–4), necroinflammation and the steatotic model based on the receiver operator curve. **a** For the advanced fibrosis model, the AUROC is 0.86 (95 % CI 0.74–0.97). **b** Accuracy of the model based on the cross-validated score and the fibrosis stage. Accuracy of the model is 82 % with a sensitivity, specificity, positive predictive value and negative predictive value of 80, 83, 71 and 89 % respectively. **c** Shows the receiver operator characteristic (ROC) curve for the necroinflammation model with an AUROC of 0.73 (95 % CI 0.57–0.89). **d** Receiver operator characteristic (ROC) curve for the steatotic model with an AUROC of 0.87 (95 % CI 0.76–0.97)

For the identification of F3–4 fibrosis, the metabolite bioprofile had an AUROC of 0.86 (95 % CI 0.74–0.97) as seen in Fig. 5. Metabolite bioprofiling facilitated the discrimination of advanced HCV fibrosis (F3-4) using a cross-validation cut off >1.39 (sensitivity 80 %, specificity 83 %, PPV 71 % and NPV 89 %) with an overall accuracy of 82 % (Fig. 2b). Similarly, the necroinflammation model, A2–3 yielded a metabolite bioprofile with an AUROC of 0.73 (95 % CI 0.57–0.89) (Fig. 5c). The steatosis model (≥33 %) produced a metabolite bioprofile with an AUROC of 0.87 (95 % CI 0.76–0.97) (Fig. 5d).

### Pathway analysis

In the fibrosis model, metabolites including amino acids, nucleic acids, and short chain fatty acids indicative of protein synthesis and catabolism as well as nitrogen metabolism were identified (Table 2; Fig. 1d–f; Additional file 1: Table S1). Alterations in metabolites were not gender-related and little or no model variance could be explained by other patient characteristics (e.g. age, body mass index). Asparagine, histidine and methionine are involved in protein biosynthetic pathways with asparagine was relatively decreased while histidine and methionine were relatively elevated with increasing fibrosis. Methionine is coupled to betaine, glycine and serine metabolism. Additionally asparagine is also involved in the ammonia-recycling pathway with glycine and glutamine. Glutamine is a member of pathways such as urea cycle, glutamate and pyrimidine metabolism.

Similar analysis of necroinflammatory disease metabolites indicated a relative up regulation of six amino acids, three dicarboxylic acids and one tricarboxylic acid (Table 2, Additional file 1: Figure S1). Pathway analysis suggests only protein biosynthesis to be impacted by this model (Additional file 1: Table S2).

On the other hand, the hepatic steatotic model (steatosis ≥33 %) included six amino acids, four dicarboxylic acids and two hydroxy acids (Table 2) over-representing several pathways related to energy and nitrogen metabolism, primarily ammonia and alanine metabolism, as well as the glucose alanine cycle (Additional file 1: Table S3, Figure S2).

It is worth noting that the pathway analysis of hepatic steatosis and necroinflammation included metabolites involved in both convergent and divergent sets of metabolic pathways. For example, in patients with steatosis several by-products of fat metabolism (e.g. acetone and 3-hydroxybutarate) and glycolysis (e.g. pyruvate and lactate) were up regulated (Fig. 3a). These compounds were not associated with fibrosis. At the same time, it is clear that nitrogen metabolism is central to all processes examined here.

## Discussion

The presence of concomitant liver fibrosis, necroinflammatory activity as well as steatosis can only be reliably confirmed by liver histological analysis. However, liver biopsy is invasive and has risk of complications [31]. In addition, liver biopsy may be limited by the size of the specimen obtained as well as sampling, intraobserver, and interobserver variability [32]. Although liver biopsy is considered the gold standard for the evaluation of the grade of necroinflammation and stage of fibrosis [4, 33, 34] many guidelines also state that transient elastography, in combination with noninvasive biomarkers (i.e., FIB-4, FibroTest), is highly useful to evaluate fibrosis in patients with chronic HCV. We propose that metabolomics is ideal to study the disease induced changes caused by any pathological process as it enables us to describe the full complement of metabolites present in in biofluids as well as tissues [3]. These metabolites represent the final downstream product of all transcriptional and translational processes within a cell. Alterations due to a disease process result in changes in groups of metabolites, which represents a pattern unique to the disease process. This metabolomic profile can be studied in detail to understand the histological changes in the tissue, which eventually leads to disease progression. For example, Cassol et al. studied the metabolomic changes in patient population with HCV and HIV co-infection. It was noted that there was a direct relationship between elevation in plasma bile acids and non-invasive markers of liver fibrosis [20]. Patients who had recorded high scores on the FIB-4 and APRI scale also had increased levels of plasma GCA and TCA. Similarly, our analysis is reflective of increased bile acid synthesis, with glycine being down regulated in the advanced fibrosis model (Fig. 1b). This is illustrated in the cholesterol conjugation pathway, where the enzyme BA-CoA:amino acid *N*-acyltransferase (BAT), amidates BA-CoA with glycine to form tertiary bile acids such as GCA and TCA [35]. Interestingly, O-acylcarnitine, which is a marker of beta oxidation of long chain fatty acid, was elevated in the necroinflammation (A2–3) group (Additional file 1: Figure S1), a finding that was supported by Cassol et al. [20], who noted a positive correlation between elevated bile acid levels and acylcarnitines.

Saito et al. [19] investigated the metabolomic response in HCV patients undergoing therapy in relation to their viral load. It was noted that patients with SVR had an elevated tryptophan levels before the initiation of treatment. Although our study did not compare pre- and post-treatment changes in metabolites, an increase in the level of tryptophan was noted in both in the advanced necroinflammation (A2–3) and steatosis group (≥33 %) analyses. On histological examination, the advanced steatosis groups tended to have advanced inflammation characteristics as well explaining the overlap. Zhang et al. were able to identify twenty distinct urinary metabolites contributing to liver disease progression [36]. Thirteen of the identified metabolites were significantly increased whereas seven were decreased. Eleven of these urinary metabolites were common to the metabolite findings in this study and included glycine, citrate, arginine, betaine, histidine, leucine, aspartic acid, succinic acid, carnitine, valine and tyrosine. In our study, citrate was differentially increased in the necroinflammation (A2–3) model and was involved in the citric acid pathway in the steatosis (≥33 %) group. Histidine was upregulated in the fibrosis (F3–4), necroinflammation (A2–3) and steatosis (≥33 %) group. Carnitine, a urinary metabolite that was decreased in the patients with disease progression, was upregulated in the necroinflammation (A2–3) and steatosis (≥33 %) group. In the necroinflammation model, it was present in the form of O-acetylcarnitine participating in the glucose-alanine oxidation of branched chain fatty acids. However, in the steatosis (≥33 %) group, it was involved in gluconeogenesis, glutamate and tryptophan metabolism. Tyrosine a urinary metabolite that was decreased in patients with disease progression was found to be up regulated in the fibrosis (F3–4) and necroinflammation (A2–3) group. In these models, it was involved in protein biosynthesis, catecholamine metabolism and phenylalanine and tyrosine metabolism pathways.

We can interpret the analysis to suggest that liver being a metabolically complex organ is representative of unique metabolomic alterations reflective of the disease process. Fibrosis, necroinflammation and steatosis of the liver are separate histological entities; the results of this pilot study suggest that we can uniquely identify metabolites that are distinctive of the three histological conditions. At the same time, certain metabolites are shared amongst three histopathological conditions, such as those in involved in urea and nitrogen metabolism (asparagine and histidine) that can be attributed to their involvement in the common metabolite pathways of amino acid, fatty acid and carbohydrate metabolism (Additional file 1: Figure S3).

Using ^1^H-NMR, we have demonstrated that serum metabolomic profiles of patients with chronic HCV differ in accordance to their histopathological state. However, larger studies will be needed to comprehensively validate the metabolic alterations associated with the histological changes in chronic HCV patients. Factors that have limited our study include a small sample size and wide gender difference in patient groups. Information regarding non-anti-viral drug use was not available. Despite these limitations, our study was stringent with respect to the standardized timing of sample collection and dietary intake (i.e., serum after a 12-h fast) as well as correlation with liver histological analysis. Although it can be argued that the metabolome is influenced by genetic variations and environmental factors, studies have indicated that a distinct metabolite signature is conserved in disease with a common etiological factor and pathophysiology [37].

## Conclusion

In this paper, we have described a proof-of-principle study that demonstrates that metabolomic profile of the chronic HCV carriers varies in accordance with the liver histopathology. Our study is able to demonstrate different metabolic profiles in association with clearcut pathology changes. It emphasizes the significance of metabolomic profiling as a promising technology for the non-invasive assessment of HCV-related liver histology. However, further experiments in the form of large-scale validation studies are required to develop our understanding of the histopathological contributions of the diseased liver site and the host, on the alterations in the metabolomic pathways, in addition to confirming the metabolomic profiles observed.

## Additional file

10.1186/s40169-016-0109-2 Additional file tables and figures.

## Abbreviations

HCV: hepatitis C virus
AUROC: area under the receiver operator curve
^1^H-NMR: proton-nuclear magnetic resonance
SVR: sustained virological response
APRI: AST to platelet ratio
HCC: hepatocellulat carcinoma
HIV: human immunodeficiency virus
F: fibrosis
A: necroinflammation
CHREB: conjoint health research ethics board
ALT: alanine-aminotransferase
AST: aspartate-aminotransferase
BMI: body mass index
NAFLD: non-alcoholic fatty liver disease
PCA: principal component analysis
OPLSDA: orthogonol partial least squared discriminant analysis
ANOVA: analysis of variance
VIP: variables importance in projection
HMDB: human metabolome database
PPV: positive predictive value
NPV: negative predictive value
FIB-4: fibrosis-4
GCA: glycolic acid
TCA: taurocholic acid
