# Supplementary material for: A quantitative metabolomics profiling approach for the noninvasive assessment of liver histology in patients with chronic hepatitis C
Source: Clin Transl Med. 2016 Aug 18;5:33. doi: 10.1186/s40169-016-0109-2 (PMC4990529; doi:10.1186/s40169-016-0109-2)
Supplement: Supplementary file 1 — 10.1186/s40169-016-0109-2 Additional file tables and figures. [file 40169_2016_109_MOESM1_ESM.docx]

**Supplementary Table 1. Biochemical pathways involved in advanced HCV fibrosis (F3-4)**

| Pathways Involved | Holm p value |
| --- | --- |
| Protein biosynthesis | 3.02E-04 |
| Ammonia recycling | 7.20E-02 |
| Glycine, Serine and Threonine Metabolism | 1.00E+00 |
| Caffeine Metabolism | 1.00E+00 |
| Aspartate Metabolism | 1.00E+00 |
| Urea Cycle | 1.00E+00 |
| Catecholamine Biosynthesis | 1.00E+00 |
| Methionine Metabolism | 1.00E+00 |
| Arginine and Proline Metabolism | 1.00E+00 |
| Intracellular Signalling through adenosine receptor A2B and adenosine | 1.00E+00 |

**Supplementary Table 2. Biochemical pathways involved in HCV necroinflammatory disease (A2-3)**

| Pathways Involved | Holm p value |
| --- | --- |
| Protein Biosynthesis | 1.92E-05 |
| Histidine Metabolism | 1.00E+00 |
| Caffeine Metabolism | 1.00E+00 |
| Phenyalanine and Tyrosine Metabolism | 1.00E+00 |
| Ammonia Recycling | 1.00E+00 |
| Glutamate Metabolism | 1.00E+00 |
| Citric acid Cycle | 1.00E+00 |
| Arginine and Proline Metabolism | 1.00E+00 |
| Catecholamine Biosynthesis | 1.00E+00 |
| Alanine Metabolism | 1.00E+00 |
| Intracellular Signalling through adenosine receptor A2B and adenosine | 1.00E+00 |

**Supplementary Table 3. Biochemical pathways involved in HCV steatosis (≥ 33%)**

| Pathways Involved | Holm p value |
| --- | --- |
| Ammonia Cycling | 8.22E-04 |
| Alanine Metabolism | 9.16E-03 |
| Glucose- Alanine Cycle | 9.26E-02 |
| Glutamate Metabolism | 3.15E-01 |
| Protein Biosynthesis | 3.65E-01 |
| Urea Cycle | 4.19E-01 |
| Citric Acid Cycle | 6.20E-01 |
| Cysteine Metabolism | 6.74E-01 |
| Malate- Aspartate Shuttle | 9.36E-01 |
| Gluconeogenesis | 1.32E-02 |
| Pyruvate Metabolism | 1.00E+00 |
| Glycine, Serine and Threonine Metabolism | 1.00E+00 |
| Intracellular Signalling through adenosine receptor A2B and adenosine | 1.00E+00 |
| Adenosine | 1.00E+00 |
| Glutathione Metabolism | 1.00E+00 |

**Supplementary Figure 1. Biochemical pathways involved in HCV advanced**

**necroinflammation (A2-3)**

**Supplementary Figure 2. Biochemical pathways involved in HCV steatosis (≥ 33%) group**. Panel (A) illustrates the metabolites that have been relatively decreased in the group. Panel (B) indicates the biochemical pathways involved in steatosis model; higher intensity with a higher association with the steatosis group. Panel (C) represents the metabolites, which have been relatively increased in the steatosis group.

**Supplementary Figure 1.**

**Supplementary Figure 2.**
